# Variability of the Martian Homopause: Insights from Global Climate Modelling and Empirical Estimates

**DOI:** 10.1007/s11214-026-01309-3

**Published:** 2026-07-16

**Authors:** Juan Alday, Francisco González-Galindo, Denis A. Belyaev, Miguel Ángel López-Valverde, Daria Kossova, Edward M. B. Thiemann, J. Scott Evans, Anna A. Fedorova, Sonal Jain, James A. Holmes, Manish R. Patel, François Forget

**Affiliations:** 1 Instituto de Astrofísica de Andalucía, CSIC, Glorieta de la Astronomía, Granada, 18008 Spain; 2 School of Physical of Sciences, The Open University, Walton Hall, Kents Hill, Milton Keynes, MK76AA UK; 3 Space Research Institute (IKI), Ulitsa Profsoyuznaya, 84/32, Moscow, 117997 Russia; 4 Laboratory for Atmospheric and Space Physics, University of Colorado Boulder, 1234 Innovation Dr, Boulder, CO 80303 CO USA; 5 Computational Physics Inc, 8001 Braddock Rd # 210, Springfield, VA 22151 VA USA; 6 Laboratoire de Météorologie Dynamique/IPSL, Sorbonne Université, ENS, PSL Research University, Ecole Polytechnique, CNRS, 4 Pl. Jussieu, Paris, 75005 France

**Keywords:** Mars atmosphere, Homopause, Eddy diffusion, Atmospheric mixing

## Abstract

The homopause marks the transition in a planetary atmosphere from turbulent mixing, which maintains a well-mixed composition, to molecular diffusion, which causes the diffusive separation of chemical species, impacting how these are distributed in the upper atmosphere and potentially escape to space. Here, we analyse simulations from the Mars Planetary Climate Model (Mars PCM) to investigate the variability of the Martian homopause in diurnal, seasonal and interannual timescales. The simulations reveal strong seasonal and latitudinal trends, with homopause altitudes peaking in the summer polar regions (∼120–130 km) and showing minimum values during the winter southern polar region (∼60–90 km). The simulations predict diurnal variations in the homopause altitude typically within 5–15 km and suggest that dust events can raise the value of the homopause altitude by 10–20 km, depending on the intensity of the event. When comparing the Mars PCM results with empirical estimates of the homopause altitude and density derived from Martian atmospheric data, we find that the model captures the overall magnitude and seasonal/latitudinal variability of the homopause, but appear to underestimate the strength of the diurnal cycle. Finally, we use the Mars PCM data to constrain the variability of the eddy diffusion coefficient, which can vary by one or two orders of magnitude across latitude and season. The derived parameterisation and variability of the eddy diffusion coefficient is suitable for use in one-dimensional models devoted to understanding seasonal difference in atmospheric photochemistry and escape.

## Introduction

In a planetary atmosphere, the homopause marks the boundary where the effects of the transition from turbulent to molecular diffusion become evident in the atmospheric composition. Below this level, in the so called homosphere, turbulence tends to keep atmospheric species well-mixed: the mixing ratio of long-lived chemical species remains approximately constant with altitude, and their number densities decrease vertically according to a common scale height determined by the mean molecular weight of the atmosphere. Above the homopause lies the heterosphere, a region where transport is dominated by molecular diffusion, which produces the diffusive separation of chemical species. In this regime, number densities decrease with altitude according to their own scale height determined by their own molecular weight. As such, the diffusive separation above the homopause has important implications for how chemical species are distributed in the upper atmosphere and for their potential escape to space.

On Mars, the first estimations of the homopause altitude were based on measurements of the Ar to N_2_ ratio from the Viking landers and revealed its location to be between 120–130 km (Nier and McElroy 1977). These early estimations of the homopause level on Mars have then been more extensively followed by measurements from the MAVEN (Mars Atmosphere and Volatile EvolutioN) mission. Mass spectroscopic measurements of Ar and N_2_ from the NGIMS (Neutral Gas and Ion Mass Spectrometer) instrument have revealed substantial variations of the homopause altitude ranging from 60 to 140 km (Jakosky et al. 2017; Slipski et al. 2018; Rao et al. 2025). These measurements also suggest that the homopause or turbopause altitude does not track a constant CO_2_ density or pressure level, suggesting variability in the dominant breaking waves and tidal modes that create turbulence in the atmosphere (Slipski et al. 2018). In addition, the measurements suggest the presence of significant interannual variability induced by solar activity and dust events (Rao et al. 2025). However, given the discrete sampling of NGIMS measurements, it is to some extent difficult to unravel the nature of the observed variability.

In addition to the mass spectroscopic measurements from MAVEN, observations from the Imaging Ultraviolet Spectrograph (IUVS) aboard this satellite (McClintock et al. 2015) have also revealed variations in the thermospheric N_2_/CO_2_ ratio that were attributed to variations in the homopause altitude (Yoshida et al. 2020). The derived homopause altitudes are generally consistent with those from NGIMS, ranging from 60–140 km, and following seasonal variations driven by the inflation and contraction of the lower atmosphere. In addition, the more complete sampling of the IUVS measurements allowed the identification of latitudinal differences in the homopause altitude during the northern summer, which decreases from about 110 km at 20^∘^N to about 90 km at 40^∘^S (Yoshida et al. 2020).

Recently, solar occultation measurements from the ExoMars Trace Gas Orbiter (TGO) have also been used to provide new insight about the Martian homopause. Using a grand average of vertical profiles from the Atmospheric Chemistry Suite (ACS), Alday et al. (2021) provided evidence for the diffusive separation of the CO_2_ isotopes above the homopause, which was derived to be located at around 100 km. Using measured CO_2_ density profiles from the same instrument, together with parameterisations of the eddy diffusion and molecular diffusion coefficients, Belyaev et al. (2022) provided estimations of the turbopause altitude (i.e., altitude at which turbulent eddy diffusion equals the molecular diffusion) at the locations of the TGO occultations across Martian Years (MY) 34 and 35. These calculations, closely related to the variations of the homopause, showed variations ranging from 90 to 100 km at aphelion to 120–130 km at perihelion.

The concept of eddy diffusion is not only useful to provide estimates of the homopause altitude, but also it is crucial to mimic the turbulent mixing in one-dimensional (1-D) models of a single atmospheric column. While these models generally offer a simplistic representation of atmospheric dynamics compared to general circulation models, which can resolve the three-dimensional (3-D) nature of planetary atmospheres, 1-D models might resolve other aspects of the atmospheric physics in greater detail (e.g., photochemistry, escape processes). On Mars, these 1-D models are commonly used for studying atmospheric escape and even aiming to understand its seasonal variability (e.g., Stone et al. 2020; Cangi et al. 2023; Kleinböhl et al. 2024). To this end, it is therefore desirable to understand the variability of the eddy diffusion coefficient to improve the parameterisation of turbulent mixing in 1-D models.

In this work, we aim to provide new insight about the variability of the homopause altitude and the eddy diffusion coefficient by utilising simulations from the Mars Planetary Climate Model (Forget et al. 1999). The Mars PCM is a general circulation model able to self-consistently simulate the whole Martian atmosphere from the surface to exosphere (González-Galindo et al. 2009), making it ideal to investigate the transition region from turbulent to molecular diffusion and its effects on the atmospheric composition. González-Galindo et al. (2009) used this model to estimate the altitude of the homopause by tracking the altitude at which the CO_2_ mixing ratio in the model was equal to 0.9. Using this methodology, they performed some preliminary analysis of the homopause variability, showing it is expected to range from 70–135 km as a function of latitude and solar longitude. Here, we aim to perform a more detailed analysis of the modes of variability of the homopause.

This article is separated into three main parts (Sects. 2, 3 and 4), explaining the methodologies and results for each of our science objectives, followed by a summary of the conclusions of this work in Sect. 5. In particular the objectives of our study are:

- Quantify the variability of the homopause altitude from the Mars PCM, focusing on both spatial and temporal variations at different timescales (i.e., diurnal, seasonal, interannual).
- Compare the predictions of the homopause characteristics from the Mars PCM with empirical data from the MAVEN/NGIMS and TGO/ACS missions, aiming not only to understand the validity of the model predictions, but also to get a better understanding of the uncertainties associated with the methodologies to analyse the mission data.
- Estimate the variability of the eddy diffusion coefficient from the Mars PCM that can be used in 1-D models.

## The Homopause Altitude from the Mars PCM

In this project, we use simulations from the Mars Planetary Climate Model to explore the variability of the homopause altitude on Mars. The Mars PCM is a unique tool for this analysis, as it models the atmosphere circulation from surface to exosphere and includes a large range of physical and chemical processes such as the effects of topography, active water and CO_2_ cycles (Montmessin et al. 2004) including the radiative effects of water ice clouds (Navarro et al. 2014), dust cycle (Madeleine et al. 2011; Wang et al. 2018; Millour et al. 2024), photochemistry (Lefèvre et al. 2004, 2021), as well as upper atmospheric processes (Angelats I Coll et al. 2005; González-Galindo et al. 2009), including the ionosphere (González-Galindo et al. 2013). The model includes, importantly, the effect of molecular diffusion in the upper atmosphere, whose relative strength with respect to other transport mechanisms defines the altitude of the homopause (Forget et al. 1999; González-Galindo et al. 2009). The model version used to build the Mars Climate Database (MCD) version 6.1 is employed here, incorporating in particular a parameterisation of the dynamical and thermal effects of the breaking of small scale gravity waves (Liu et al. 2023), though not their effects on the mixing (Liu et al. 2025). The horizontal resolution of the model is 5.625^∘^ (longitude) × 3.75^∘^ (latitude), with 73 vertical levels with higher vertical resolution close to the surface.

The simulations used in this study are run assuming different scenarios to investigate the variability of the homopause altitude at different scales. Specifically, simulations run using the “climatological scenario”, which includes dust loading levels representative of a baseline typical Mars year, as well as average solar EUV conditions, are used to analyse the latitudinal, seasonal and diurnal variability. On the other hand, the interannual variability is evaluated by comparing the predicted variability of the homopause using specific scenarios from MY32 to MY36 that incorporate the observed day-to-day variability of the dust load in the lower atmosphere (Montabone et al. 2015, 2020), as well as the daily variation of the EUV solar flux (González-Galindo et al. 2013).

### Estimation of the Homopause Altitude from the Mars PCM

**Fig. 1:**
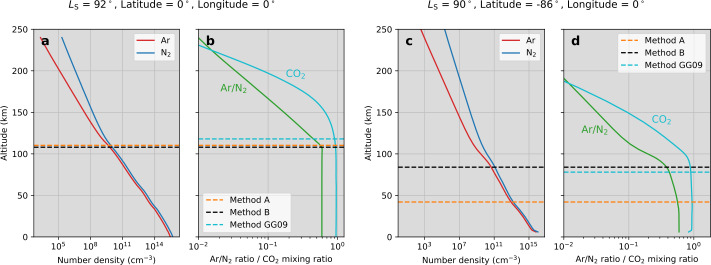
Summary of the methodology used to estimate the homopause altitude using the Mars PCM at two distinct locations. For each location, the left panels (a,c) show the number densities of Ar and N_2_ from the model, while the right ones (b,d) show their density ratio (green solid line), together with the CO_2_ mixing ratio (cyan solid line). The dashed orange and black horizontal lines indicate the homopause altitudes derived from the Ar/N_2_ ratio using Methods A and B, respectively (see text). The dashed cyan horizontal line represents the homopause altitude derived from the CO_2_ mixing ratio following González-Galindo et al. (2009)

To estimate the altitude of the homopause from the simulations, we use Ar and N_2_ densities – the ratio of these two species, which are largely chemically inert, is approximately constant in the homosphere, while it varies due to diffusive separation above the homopause, given their different molecular weight. Specifically, given the heavier molecular weight of Ar (m = 40) with respect to N_2_ (m = 28), the Ar/N_2_ will decrease with increasing altitude above the homopause level (see Fig. 1a). Based on this behaviour of the vertical variability of the Ar/N_2_ ratio, we estimate the altitude of the homopause using two different methodologies:

- Method A: Altitude at which the Ar/N_2_ ratio is 5% lower than that between 20–30 km. This is similar to the approach of González-Galindo et al. (2009), who tracked variations in the homopause altitude using the level at which the CO_2_ mixing ratio is approximately 5% lower than in the lower atmosphere (i.e., CO_2_ mixing ratio of 0.9).
- Method B: Altitude at which we find the minimum of $\partial ^{2}R/\partial z^{2}$ in the profile, being R the Ar/N_2_ ratio and z the vertical coordinate. Above this point, we generally find that R varies approximately linearly in logarithmic scale, consistent with a transport regime dominated by molecular diffusion.

**Fig. 2:**
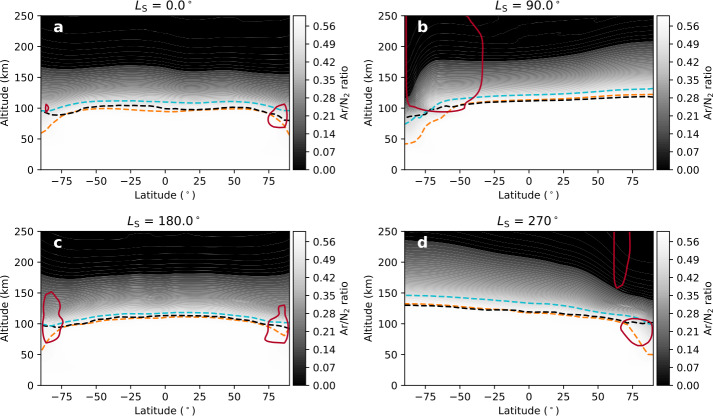
Comparison of the two methods used to estimate the homopause altitude from the Mars PCM. The four panels show the zonal mean of the Ar/N_2_ ratio during the equinox and solstice periods. The orange and black dashed lines represent the homopause altitudes derived from the Ar/N_2_ ratio using Methods A and B, respectively. The dashed cyan horizontal line represents the homopause altitude derived from the CO_2_ mixing ratio following González-Galindo et al. (2009). The red contours indicate the regions in the model where the downward winds exceed 0.35 m/s

We find that both methods provide similar estimations of the homopause altitude for most seasons and locations (see Fig. 2). However, we do find some notable differences primarily in the polar regions (>60^∘^), where the estimations from both methods can differ by several tens of kilometres due the Ar/N_2_ ratio not following a constant behaviour with altitude below the homopause (see Fig. 1b,d). Specifically, we find that the locations and seasons where these two methods differ correlate with the presence of strong vertical downward winds at high latitudes (see Fig. 2 and Fig. 18). Strong downward winds may also be found at other times and locations throughout the year (Bougher et al. 2015). Under these conditions, downwelling of low Ar/N_2_ air from the upper atmosphere to lower altitudes breaks the condition of well-mixed ratios below the homopause, complicating the estimation of the homopause altitude with Method A.

Upward winds below the homopause may produce the opposite effect by transporting well-mixed air to higher altitudes, effectively increasing the homopause altitude. However, upward winds around the homopause region are generally much weaker than downward winds and are therefore expected to have only a relatively minor effect (see Fig. 18). Taking these considerations into account, we consider that Method B provides a more realistic representation of the altitude region above which molecular diffusion starts dominating, and therefore select it as our preferred method for estimating the homopause altitude on Mars from the model.

González-Galindo et al. (2009) also investigated variability in the homopause altitude using an earlier version of the Mars PCM, tracking the level at which the CO_2_ mixing ratio equals 0.9. Figures 1 and 2 compare this approach with the methodology adopted here based on the Ar/N_2_ ratio. Both methods show similar variability in the homopause altitude; however, the CO_2_-based estimates are on average 5–10 km higher than those derived from the Ar/N_2_ ratio, consistent with González-Galindo et al. (2009). Comparison of the vertical profiles indicates that the Ar/N_2_ ratio exhibits a sharper transition at the homopause, which facilitates its numerical identification in the simulations. We therefore consider the Ar/N_2_ profiles to provide a more robust estimate of the homopause altitude in the Mars PCM and adopt this as our preferred method. All subsequent figures show homopause altitudes derived from the Ar/N_2_ ratio using Method B.

### Variability of the Homopause Altitude

In this sub-section, we analyse the variability of the homopause altitude as predicted by the Mars PCM in different timescales, including diurnal, seasonal and interannual variations.

#### Seasonal Variations

**Fig. 3:**
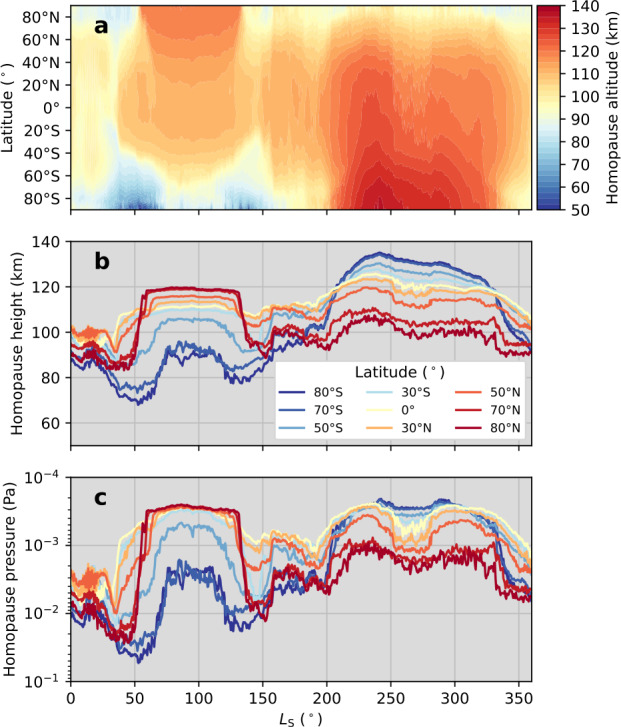
Seasonal and latitudinal variability of the homopause altitude. a) Evolution of the homopause altitude as a function of solar longitude and latitude, after being averaged across different longitudes and local times. b) Evolution of the homopause altitude in panel a at distinct latitudes, following the legend. c) Evolution of the homopause pressure at distinct latitudes, following the legend in panel b

Figure 3a,b shows the diurnally and longitudinally-averaged seasonal evolution of the homopause altitude as predicted from the Mars PCM, which reveals significant variations of this altitude. Figure 3c shows the variations in homopause pressure, which exhibit large changes tightly correlated with altitude, as discussed later in Sect. 2.3. These variations in the homopause pressure level suggest that the changes in homopause altitude are not solely driven by simple atmospheric expansion and contraction, which do not drive variations in the homopause pressure, but rather by variations in the mixing strength.

The highest homopause altitudes are found at the high latitudes of each hemisphere during the summer season, with values of approximately 120 km at >60^∘^N during $L_{\mathrm{S}} =60$–$130^{\circ}$, and approximately 130 km at >60^∘^S during $L_{\mathrm{S}} =210$–$320^{\circ}$. During these seasonal periods we also find the largest latitudinal variability, with the homopause altitude decreasing from the summer to the winter hemispheres: between $L_{\mathrm{S}} = 60$–$130^{\circ}$, the altitude of the homopause decreases from ∼120 km at >60^∘^N to 105–110 km between 60^∘^S–60^∘^N and to 60–90 km at >60^∘^S; between $L_{\mathrm{S}} = 210$–$320^{\circ}$, the homopause altitude decreases from ∼130 km at >60^∘^S to 110–120 km between 60^∘^S–60^∘^N and to 100–110 km at >60^∘^N.

During the equinox periods, the latitudinal distribution of the homopause altitude is instead more symmetric around the equator: during the spring equinox in the northern hemisphere ($L_{\mathrm{S}}\sim 0^{\circ}$), the homopause altitude lies at 90–100 km between 60^∘^S–60^∘^N and slightly decreases to ∼80–90 km at higher latitudes; during the autumn equinox in the northern hemisphere ($L_{\mathrm{S}}$ ∼ 180^∘^) the homopause altitude is about 10 km higher than during the spring equinox, ranging from 100–110 km between 60^∘^S–60^∘^N and ∼90–100 km at latitudes >60^∘^.

These seasonal patterns reflect changes influenced by the general circulation of the atmosphere. During the solstices, the atmospheric circulation is dominated by a single Hadley cell centred at a latitude of ∼30^∘^ of the summer hemisphere. During the southern hemisphere summer solstice (i.e., $L_{\mathrm{S}} = 270^{\circ}$), the circulation strengthens by the closer proximity to perihelion and thus increased solar forcing, the higher dust optical depths, and the orography of the Martian surface, all of which contribute to an increased circulation and enhanced atmospheric mixing (Haberle et al. 1993; Richardson and Wilson 2002). This, together with the larger expansion of the atmosphere close to perihelion, explains the higher homopause altitudes found during southern summer with respect to northern summer. The similar homopause pressures in the summer polar latitudes during both solstices suggest that the differences in homopause altitudes in these regions (>60^∘^N at $L_{\mathrm{S}} = 90^{\circ}$ and >60^∘^S at $L_{\mathrm{S}} = 270^{\circ}$) are largely driven by atmospheric expansion during perihelion. On the other hand, in the winter high latitudes, differences in both homopause altitudes and pressures reveal the influence of the stronger atmospheric circulation during perihelion and the enhanced mixing.

The circulation during the equinox periods (i.e., $L_{\mathrm{S}} = 0$, 180^∘^) is instead driven by two roughly symmetric Hadley cells. The strength of the circulation during equinox is much weaker than during solstice (Haberle et al. 1993), which explains the overall lower homopause altitudes during these periods. Homopause altitudes around the northern spring equinox (i.e., $L_{\mathrm{S}}$ ∼ 0^∘^) are lower on average by about 10 km with respect to the northern autumn equinox period (i.e., $L_{\mathrm{S}}$ ∼ 180^∘^). This is likely related to the greater Mars–Sun distance around $L_{\mathrm{S}}$ ∼ 0^∘^ (1.56 AU) than around $L_{\mathrm{S}}$ ∼ 180^∘^ (1.47 AU), which translates in differences in the solar heating.

Periods between equinoxes and solstices are characterised by strong and sudden changes in the homopause altitude. For example, there is a strong increase in the homopause altitudes between $L_{\mathrm{S}} \sim $ 30–70^∘^ and $L_{\mathrm{S}} \sim $ 200–230^∘^, and decrease between $L_{\mathrm{S}} \sim $ 120–150^∘^ and $L_{\mathrm{S}} \sim $ 330–350^∘^. These periods are characterised by strong modifications in the circulation pattern, when it changes between equinoctial to solstitial configurations, or vice-versa. These changes in the circulation and illumination conditions also generate planetary waves that produce substantial diurnal variability, as later discussed in Sect. 2.2.2.

The variability in the homopause level can also be influenced by the effects of gravity wave breaking. The variations in the homopause level in our simulations, which include a parameterisation for the dynamical and thermal effects induced by breaking of small-scale gravity waves, are consistent with those from González-Galindo et al. (2009), which did not include any parameterisation of non-orographic gravity waves. This suggests that, in both cases, the variability of the homopause is primarily controlled by resolved-scale atmospheric circulation patterns. Nevertheless, it must be noted that our simulations do not explicitly include the effects of gravity wave breaking on turbulent mixing processes (Liu et al. 2025), which may also influence the altitude at which the homopause is set and contribute to additional variability.

#### Diurnal Variations

**Fig. 4:**
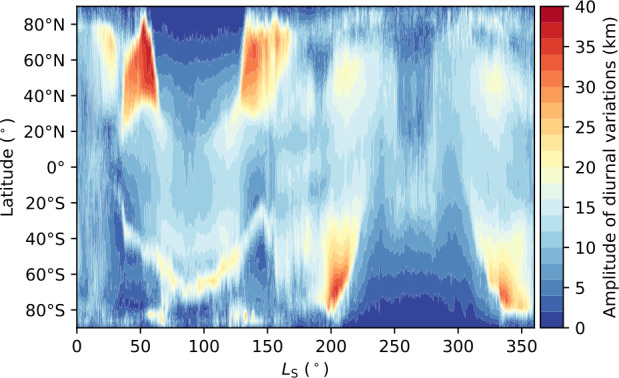
Amplitude of the diurnal variability of the homopause altitude. The contours represent, as a function of latitude and solar longitude, the difference between the maximum and minimum homopause altitudes within each sol

**Fig. 5:**
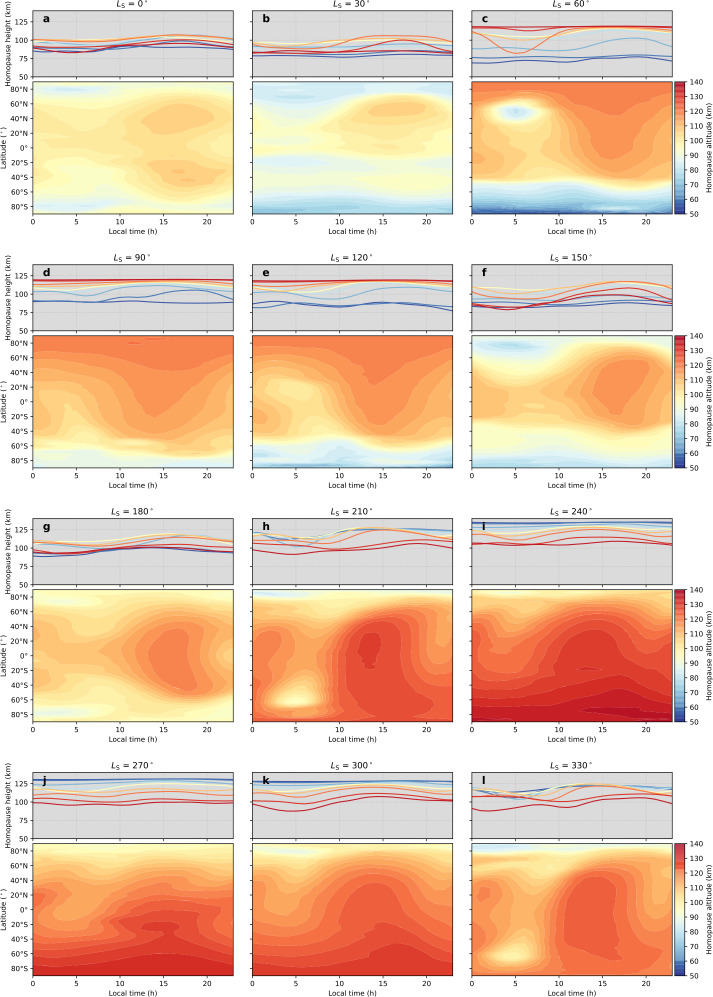
Examples of the diurnal cycle of the homopause altitude at different solar longitudes. The contour plots show the latitude vs local time variability of the homopause altitude at some selected times of the year, indicated by the solar longitude above each panel. The panels on top show similar information, but now shown as line plots coloured by the latitude, following the legend in Fig. 3

Apart from the seasonal variability, we explore the variability of the homopause altitude as a function of local time. To get a first insight, Fig. 4 shows the maximum change in the homopause altitude occurring within each sol. This quantity is calculated by identifying, for each latitude and sol within a year, the maximum and minimum homopause altitudes during each sol and computing their difference, thus providing a measure of the strength of the diurnal variability. In addition, Fig. 5 presents the full diurnal cycle at selected solar longitudes, complementing the information shown in Fig. 4. These figures show that the diurnal variability strongly depends on latitude and season and that, despite differences in absolute values, the latitudinal and diurnal variability of the homopause altitude remains broadly symmetric around the solstices.

During the northern hemisphere summer solstice ($L_{\mathrm{S}} = 90^{ \circ}$, see Figs. 3a and 5d), the highest homopause altitudes are found in the summer polar region (>60^∘^N), where the diurnal variability is minimal due to the nearly constant illumination conditions (i.e., polar day). Across the rest of the summer hemisphere, the diurnal variability remains weak, with amplitudes below 5 km and a gradual decrease from the daytime peak at around 14–15 h. In the low-to-mid latitudes of the winter hemisphere (0^∘^–40^∘^S), the homopause remains elevated for most of the day, from around 9–10 h until 2–3 h in the morning, followed by a sharper decrease between 2–3 h and 8–9 h, with overall variations of around 10–15 km. The strongest variations during this season are found within a narrow band at 60–70^∘^S (∼15–20 km), where the homopause rapidly decreases after the evening peak at ∼18–19 h. At latitudes of 70–90^∘^S, the variability again becomes minimal because of the persistent dark conditions associated with polar night.

The diurnal variability during the southern summer solstice ($L_{ \mathrm{S}} = 270^{\circ}$, see Figs. 3a and 5j) resembles that of the northern summer solstice, with weak variability in the summer polar region (>60^∘^S) and at high latitudes of the winter hemisphere (>70^∘^N). The strongest variations are found between 0–20^∘^S (∼10 km), where the homopause decreases smoothly from the daytime peak at 15 h to minimum altitudes at 4–5 h. At the mid-latitudes of the winter hemisphere (30–60^∘^N), the amplitude of the diurnal variations remains around ∼5 km and shows two maxima at 14–15 h and 2–3 h, together with minima at 5–7 h and 20–22 h. This behaviour is consistent with the close relationship between atmospheric aerosol loading and the amplitude of semidiurnal tides observed in surface pressure and atmospheric temperature measurements by different missions and simulated by numerical models (Banfield et al. 2000; Kleinböhl et al. 2013).

During the equinoxes ($L_{\mathrm{S}} = 0$, 180^∘^, Fig. 5a,g), the sub-solar latitude is centred near the equator and the atmosphere is approximately symmetric between hemispheres. At low and mid-latitudes (60^∘^S–60^∘^N), the homopause altitude generally peaks between 16–18 h and reaches minimum values between 5–7 h. At $L_{\mathrm{S}} = 0^{\circ}$, the global maxima occur near ∼40^∘^N and ∼40^∘^S rather than at the equator, suggesting an important role of atmospheric dynamics in modulating the homopause altitude. At $L_{\mathrm{S}} = 180^{\circ}$, these latitudinal structures are less pronounced during the daytime peak (16–18 h), although enhancements still appear near ∼30^∘^N and ∼30^∘^S between 20 h and 3 h. These latitudes also show the largest diurnal variations (∼15–20 km) compared with the equatorial region (∼10–15 km).

The periods between equinoxes and solstices show a more complex latitudinal and diurnal structure, likely associated with rapid transitions in atmospheric circulation between solstice and equinox configurations. These periods host the largest diurnal variations (i.e., $L_{\mathrm{S}}\sim $50, 150, 210, and 330^∘^), with amplitudes increasing to around 30–40 km at latitudes of 40–70^∘^ (in the northern hemisphere at $L_{\mathrm{S}}\sim $50 and 150^∘^, and in the southern hemisphere at $L_{\mathrm{S}}\sim $210 and 330^∘^), as shown in Fig. 4. These large amplitudes appear to result from a rapid nighttime collapse of the homopause altitude between 2–3 h and 7–8 h, with minimum values occurring near ∼5 h. Other latitude bands display evidence of higher-order diurnal variability, highlighting the role of tides in modulating the homopause altitude (see Fig. 5e,h).

Our simulations therefore show that, to first order, the homopause altitude typically peaks during the daytime (12–18 h) and decreases during the night, reaching minimum values around 5–7 h. More complex patterns, however, indicate that the homopause altitude on Mars is modulated by changes in illumination conditions, tides, dust loading, and atmospheric dynamics. To first order, this behaviour is consistent with the analysis of NGIMS measurements (Slipski et al. 2018; Rao et al. 2025), which show the highest altitudes around noon and minimum altitudes near 5 h. The comparison with NGIMS data is discussed further in Sect. 3.1.

#### Interannual Variations

**Fig. 6:**
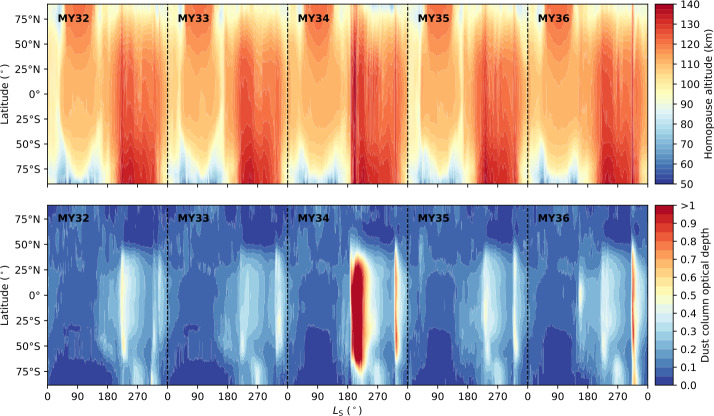
Variations of the homopause altitude and dust column optical depth from MY32-36. Top) Diurnally-averaged homopause altitude from the Mars PCM as a function of latitude and solar longitude. Bottom) Diurnally-averaged dust column optical depth from the maps of Montabone et al. (2015, 2020)

The simulations from the Mars PCM also allow the adaptation of scenarios specific for different Martian years, allowing the analysis for interannual variability of the homopause and how it is affected by other meteorological parameters, such as the dust abundance and the solar activity. Figure 6 shows the seasonal and latitudinal variability of the homopause altitude across MY32-36, together with the evolution of the dust column optical depth for each specific year (Montabone et al. 2015, 2020). The simulations reveal that the seasonal variations described in Sect. 2.2.1 are generally repeated year after year. However, events such as dust storms can have a direct impact on the variability of the homopause altitude.

Around $L_{\mathrm{S}}\sim 300$–$330^{\circ}$, most Martian years host the presence of a type C dust storm, where the dust optical depth rises in the middle and low latitudes for a period of around 3^∘^–15^∘^ of solar longitude (Kass et al. 2016). The strength of these events varies from year to year and was especially strong during MY34 and MY36 (see Fig. 6). Similarly, some stochastic dust events may increase the dust optical depth over the entire planet – these are known as Global Dust Storms (GDS) and one of these occurred in MY34. The simulations from the Mars PCM predict a direct response of the homopause altitude to these events, raising in altitude by about 10–20 km depending on the intensity of the event.

**Fig. 7:**
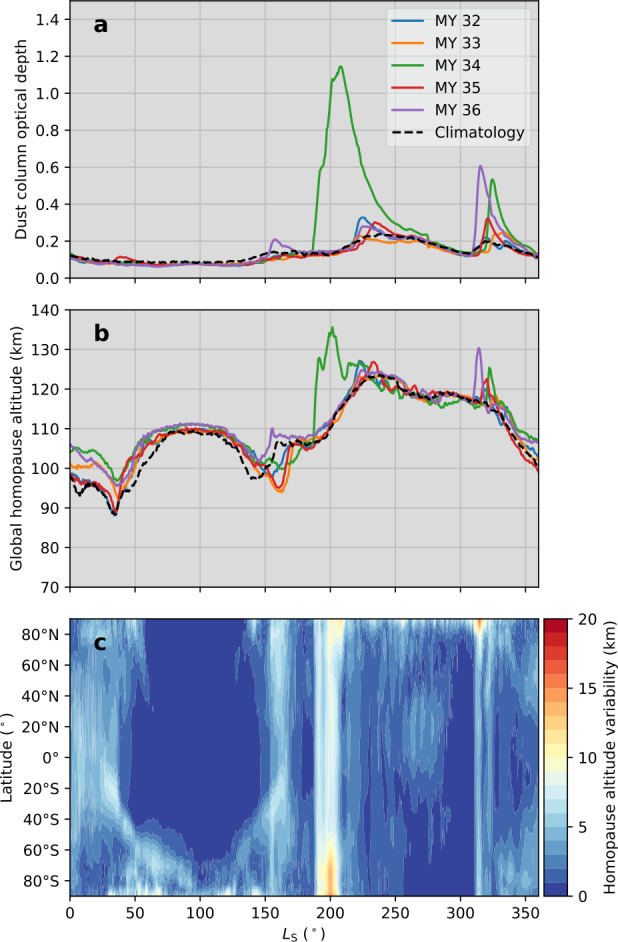
Overview of the interannual variability of the homopause altitude from the Mars PCM. Top) Diurnally and spatially-averaged dust column optical depth across a full year. Middle) Diurnally and spatially-averaged homopause altitude. Bottom) Standard deviation of the diurnally-averaged homopause altitude across MY32-36

Apart from the response of the homopause altitude to dust storm events, we can explore its interannual variability at other times of the year. Figure 7 suggests that, aside from dust-induced responses, interannual variability peaks around the equinox periods ($L_{ \mathrm{s}}\sim 0$–$40^{\circ}$ and $L_{\mathrm{S}}\sim 150$–$190^{\circ}$) at all latitudes, and in the southern polar region (>60^∘^S) during the first half of the year, with a magnitude of around 5–10 km. In contrast, homopause altitudes in the summer hemispheres (i.e., northern and southern hemispheres during first and second halves of the year, respectively) are much more repeatable from year to year, showing little evidence of interannual variability. This may indicate a difference in the processes governing the variability of the homopause altitude: during the solstice periods the stronger atmospheric circulation, more repeatable from year to year, defines the characteristics of the homopause altitude, whereas the homopause is more affected by planetary waves and other phenomena during the equinox periods, when the circulation is relatively weaker (Haberle et al. 1993).

### Homopause Characteristics

**Fig. 8:**
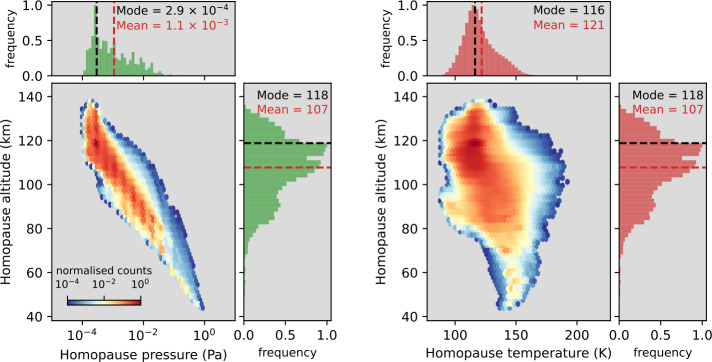
Relation of the homopause altitude with other atmospheric parameters. The panels here show 2-D histograms of the relation between the homopause altitude with respect to the pressure (left) and temperature (right). The colour of the 2-D histograms shows the normalised frequency of the occurrences (in log scale), following the colourbar on the left panel. In addition, the 1-D representation of these histograms for each of the parameters is shown at the sides of each panel, highlighting the mode and mean of each distribution

The analysis presented in the previous subsection reveals that the homopause altitude is expected to follow significant spatiotemporal variations in different timescales. In this section, we aim to get insight about how the variations in this altitude relate to other atmospheric parameters. Figure 8 shows histograms and correlation plots of the homopause altitude with respect to the pressure and temperature at this level, which can be used to infer some insight about the properties of the atmosphere at the homopause.

The vast majority of homopause altitudes range from 70–130 km, with the mode and mean of the distributions being found at 118 and 107 km, respectively. The homopause altitude may fall behind this lower bound on rare occasions, mainly at the high latitudes in the southern winter hemisphere (see Fig. 3). The relation with the pressure evidences that the homopause does not sit at a fixed pressure level, but can range over several orders of magnitude ($10^{-4}$ to $10^{-1}$ Pa), with the mode and mean of the distribution at 0.3 mPa and 1.1 mPa. This is consistent with results from MAVEN data, which suggest that the homopause altitude does not sit at a constant CO_2_ density level (Slipski et al. 2018; Yoshida et al. 2020) and which has implications for our understanding of the homopause altitude. Specifically, homopause altitude variations caused by the inflation and contraction of the lower atmosphere due to changing of solar irradiance sit at a fixed pressure level, but changes in the homopause altitude caused by the varying turbulent mixing (i.e., eddy diffusion) from wave braking drive variations in the homopause pressure too (Slipski et al. 2018; Yoshida et al. 2020).

The simulations from the Mars PCM suggest a much milder correlation between the homopause altitude and temperature. The vast majority of the homopause temperatures lie within 100–150 K, with a mode and mean of the distribution centred at 116 and 121 K, respectively. While the correlation with the temperature is not as strong as with pressure, we do find some synchronous variability in both parameters: the mean of the temperature distribution for the homopause altitude points above 110 km is 115 K and it increases to 127 K for 80 < h < 110 km, and 142 K for homopause altitudes below 80 km.

## Comparison of the Model Predictions with Empirical Estimates

In the previous section, we have analysed the variations of the homopause altitude from the Mars PCM, aiming to get some insight about its spatiotemporal variability and its relation with other atmospheric parameters. In this section, we want to compare the predictions of the Mars PCM with estimates of the homopause characteristics inferred from empirical data, aiming to not only assess the validity of the model, but also to better understand the uncertainties and biases of the empirical estimations. Here, we compare the model predictions against empirical estimates from MAVEN/NGIMS and TGO/ACS.

### Comparison Against MAVEN/NGIMS

In this sub-section, we compare the predictions of the homopause characteristics from the Mars PCM against empirical estimates using mass spectrometric data from the NGIMS instrument aboard NASA’s MAVEN mission, following a methodology similar to that used in previous investigations (Jakosky et al. 2017; Slipski et al. 2018; Rao et al. 2025). In particular, we use the Level 2, version 08, revision 01 data, which provides number densities of different chemical species measured during each orbit (Elrod et al. 2014). For each orbit, NGIMS produces one dataset containing measurements from both the inbound and outbound legs as MAVEN approaches and departs from periapsis. The altitude of the periapsis point has substantially changed across the mission lifetime. Here, we include only observations with periapsis altitudes below 170 km to avoid an excessive extrapolation of the Ar/N_2_ profiles to the lower homopause altitudes, covering measurements from February 2015 ($L_{\mathrm{S}} \sim 280^{\circ}$ in MY32) to June 2020 ($L_{\mathrm{S}} \sim 210^{\circ}$ in MY35). After this date, the periapsis altitude of MAVEN’s orbit was raised and has not met this criterion since. Overall, the extent of the dataset analysed here is equivalent to that used by Rao et al. (2025).

**Fig. 9:**
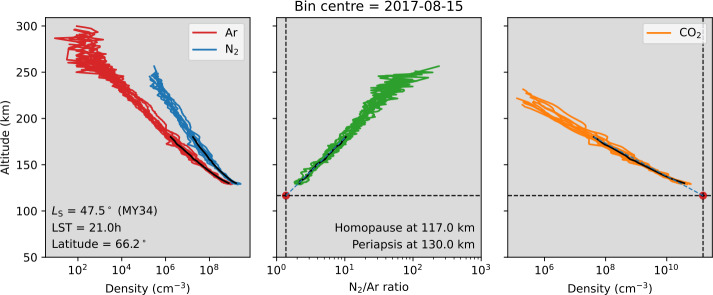
Summary of the methodology to estimate the homopause altitude and CO_2_ density from the MAVEN/NGIMS measurements. The left panels show all the Ar (red) and N_2_ (blue) profiles measured within a two-day bin centred on 2017-08-16. The left panel shows the Ar and N_2_ number densities profiles (red and blue lines). The middle panel shows the N_2_/Ar ratio (green lines) as well as the derived homopause altitude by extrapolation of the ratio to the lower atmospheric value. The right panel shows the measured CO_2_ densities (orange) line. In all panels, the black solid lines represent the averaged profiles in a 50-km region above the periapsis point

To estimate the homopause altitude and CO_2_ density from the data, we combine the Ar, N_2_ and CO_2_ density profiles measured during the inbound legs of the orbit into two-day bins. Within these bins, although the periapsis point shifts across a wide range of longitudes, the latitudes and local times remain essentially constant. Following previous works (Jakosky et al. 2017; Slipski et al. 2018; Rao et al. 2025), we estimate the homopause altitude by fitting a linear relation (in logarithmic scale) to the measured N_2_/Ar ratio profile from the periapsis to 50 km above, extrapolating it downward until it matches the lower-atmosphere value derived from Curiosity Rover measurements (N_2_/Ar = $\frac{0.0279\pm 0.0005}{0.0208\pm 0.0002} = 1.341\pm $ 0.027; (Franz et al. 2017)) (see Fig. 9). Once the homopause altitude is estimated, we perform a similar calculation to estimate the CO_2_ density at this level: the measured CO_2_ density profile in the same altitude range is fitted using a linear function in logarithmic scale and is extrapolated downward to the inferred homopause level.

The methodology described above yields estimates of the homopause altitude and CO_2_ density for each bin, which is interpreted to be representative of the location and local time at the periapsis. However, it remains difficult to evaluate the uncertainty of these measurements. In particular, one source of uncertainty arises from the sampling of the NGIMS measurements, which combines vertical and horizontal variability that may introduce biases for the downward extrapolation of the N_2_/Ar ratio. In addition, even if the NGIMS measurements were all made in a single atmospheric column, the extrapolation of the measurements from the periapsis altitude to the homopause altitude can be compromised if there are substantial changes in the scale height between these two altitudes. To get an estimate of the uncertainties in the NGIMS estimations of the homopause characteristics, we take advantage of the simulations from the Mars PCM. Specifically, we extract Ar, N_2_, and CO_2_ densities from the model at the times and locations of the NGIMS measurements using the Mars PCM calculations for each specific Mars year, apply the same methodology to derive the homopause characteristics, and compare the results with the model-derived homopause altitude and density at periapsis (i.e., the nominal homopause altitudes from the model presented in Sect. 2). The differences between the two provide estimates of the biases and uncertainties in the NGIMS-based retrievals and allow us to conduct a more robust and comprehensive analysis of the dataset.

**Fig. 10:**
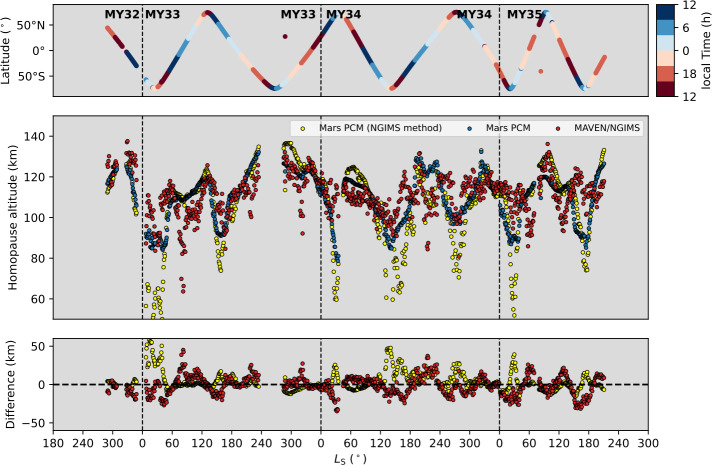
Overview of the comparison of homopause altitudes from the Mars PCM and MAVEN/NGIMS. The top panel shows the latitude and local time of the NGIMS measurements at periapsis. The middle panel shows the estimated homopause altitudes with MAVEN/NGIMS in each bin (red), the homopause altitude from the Mars PCM at the periapsis point as explained in Sect. 2.1 (blue), and the estimated homopause altitudes when applying the NGIMS methodology to the PCM data (yellow). The bottom panel shows the differences of the homopause altitudes from the Mars PCM (blue points in middle panel) with respect to the homopause altitudes estimated using the NGIMS methodology in the real NGIMS data (red points) and simulated PCM data (yellow points)

Figure 10 provides an overview of the comparison results as a function of time. The magnitude and variability of the homopause altitude inferred from the MAVEN/NGIMS data are consistent with previous studies, lending confidence to the validity of our calculations when working with the NGIMS data (Rao et al. 2025; Slipski et al. 2018). When applying the same methodology to the data from the Mars PCM (yellow points in Fig. 10), we find that this methodology can overall reproduce the variability of the homopause altitude from the model. However, in several instances, primarily in the polar regions (>60^∘^), we find large deviations between the modelled homopause altitudes and the ones derived using the NGIMS sampling and methodology (but modelled data). These deviations are mostly caused by the not-well-mixed condition of the N_2_/Ar ratio: the ratio of these two species at the homopause in the polar region is not the one representative of the lower atmosphere, since it has been mixed with air with high N_2_/Ar downwelled from the upper atmosphere (see Sect. 2.1). Therefore, when extrapolating to a fixed value given by Curiosity, we systematically underestimate the altitude of the homopause. Interestingly, these large deviations in the polar regions are less pronounced in the NGIMS data than in the synthetic tests and may even appear anti-correlated. In fact, the agreement between the Mars PCM and NGIMS-derived homopause altitudes during these periods is, to some extent, better than when applying the NGIMS methodology to the PCM data, suggesting that the winds in the downwelling branches of the Hadley cell in the model may be too strong. Nevertheless, these deviations underscore the need for caution when interpreting estimates of the homopause altitude in the winter polar regions.

**Fig. 11:**
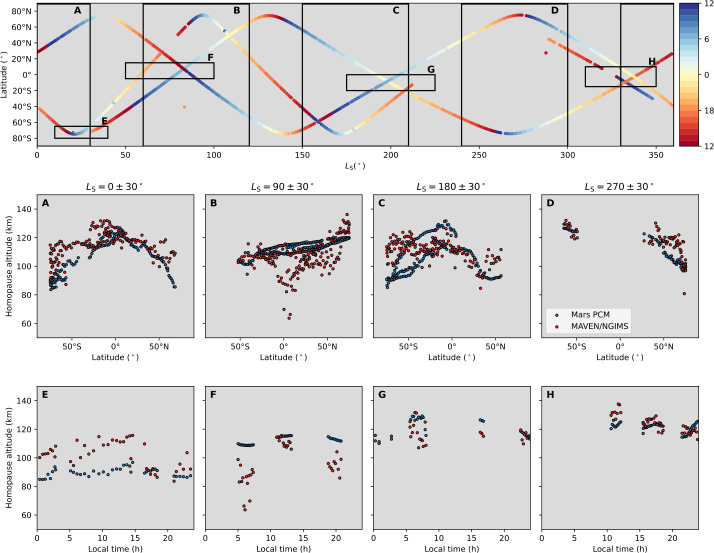
Comparison of the latitudinal and diurnal trends of the homopause altitude modelled with the Mars PCM and estimated using data from MAVEN/NGIMS. The top panel shows the latitude and local time of the NGIMS measurements at periapsis. The four middle panels (A–D) show the latitudinal variability of the homopause altitudes from the Mars PCM calculated as explained in Sect. 2.1 (blue) and MAVEN/NGIMS (red) during $L_{\mathrm{S}}$ periods of ±30^∘^ centred at the solstices and equinoxes. The four bottom panels (E–F) show the diurnal trends at four specific $L_{\mathrm{S}}$ and latitudinal ranges highlighted in the top panel

Overall, Fig. 10 suggests that the magnitude and variability of the homopause altitude modelled with the Mars PCM is consistent with the NGIMS measurements: the mean homopause altitude and standard deviation from the two datasets is 110 ± 12 km and 110 ± 11 km, respectively. This variability arises from both spatial and temporal variability on different timescales. To further explore the origin of this variability and how the two datasets compare, Fig. 11 presents the data as a function of latitude, season, and local time.

During the spring equinox in the northern hemisphere ($L_{\mathrm{S}} \sim 0^{\circ}$), the homopause altitude peaks in the equatorial region and decreases towards the poles (Fig. 11A). At latitudes <60^∘^S, the NGIMS data suggest more variability than the model, which appears to be related to the local time. Specifically, both the model and data reveal homopause altitude of around 90 km after 15 h (Fig. 11E). However, while both predict the peak of the homopause altitude between 12–15 h, the homopause altitudes from NGIMS (∼110–120 km) are higher than predicted in the model (∼90–100 km), and this shift of around 20 km is also observed for most of the nighttime. This suggests that the amplitude of the local time variations from the Mars PCM in this period is underestimated with respect to the observations.

During the northern summer solstice ($L_{\mathrm{S}} \sim 90^{\circ}$), the homopause peaks at the north pole and slightly decreases with decreasing latitude (Fig. 11B). The PCM simulations suggest that below ∼50–60^∘^S the homopause altitude should significantly decrease, but unfortunately the NGIMS coverage does not include measurements in the southern polar region during this season. The NGIMS-derived altitudes around the equator (15^∘^S–15^∘^N) appear to show two distinct populations, one centred at around 105–110 km, and another one at 80–90 km. When analysing the diurnal variability in this region (Fig. 11F), the NGIMS dataset reveals a strong decrease of the homopause altitude from noon (∼110 km) towards the morning and evening (∼80–90 km). On the other hand, the Mars PCM predicts much milder variations as a function of local time, suggesting that the strength of the diurnal cycle is underestimated in the model. This underestimation of the diurnal trends in the Mars PCM is also observed in other parameters, such as the temperature, where comparisons with MAVEN datasets in the upper atmosphere highlight the inability of the PCM to reproduce the amplitude of the diurnal variations (González-Galindo et al. 2026).

**Fig. 12:**
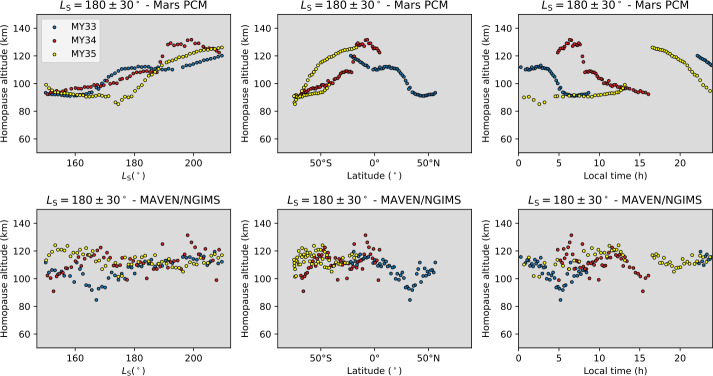
Variability of the homopause altitudes from the Mars PCM and MAVEN/NGIMS during $L_{\mathrm{S}}$ = 150–210^∘^, which encompassed the GDS in MY34. The top panels show the modelled homopause altitudes at the periapsis points of the MAVEN/NGIMS observations, while the bottom panels show the homopause altitudes estimated with the NGIMS data

During the autumn equinox ($L_{\mathrm{S}} \sim 180^{\circ}$), the comparison between model and data can become complex, as it encompasses potential interannual variability triggered by the Global Dust Storm in MY34 (Fig. 11C,G). Figure 12 shows similar information as Fig. 11 for this period, but now colouring the data by MY, so that we can investigate the potential interannual variability. The model predicts a clear increase of the homopause altitude of around 10 km after $L_{\mathrm{S}} \sim 180$–$190^{\circ}$ in MY34. However, when comparing the model predictions of the homopause altitude during this period between MY34 and MY35, they are comparable. This is because the NGIMS coverage in MY35 is centred around 15–20 h, when the effect of the diurnal cycle peaks. When analysing the NGIMS data, we observe the highest altitudes during the MY34 GDS period, but these altitudes are overall not much higher than during the other MYs. Our interpretation is that the MY34 GDS likely had an impact in the homopause altitude, but this impact is still subject to comparable latitudinal and diurnal variability, which complicates identifying the impact of the GDS in the NGIMS data.

In their analysis of the NGIMS data, Rao et al. (2025) highlighted that homopause estimates during the MY34 GDS increased during the growth phase and decreased during the declining phase, but also exhibited significant fluctuations. These fluctuations were attributed to enhanced gravity wave activity during the dust storm (Kuroda et al. 2020; Leelavathi et al. 2020), which can induce local variations in homopause altitude. In our analysis, the scatter of the retrieved homopause estimates during the GDS appears comparable to that observed during other periods (see Fig. 10), making it difficult to unambiguously associate the observed variability with enhanced gravity wave activity during the GDS. These fluctuations are not predicted by the Mars PCM simulations, which predict a substantial overall increase in homopause altitude driven by increased heating rates in the middle atmosphere. However, as noted previously, our simulations do not explicitly include the effects of gravity wave breaking on turbulent mixing processes (Liu et al. 2025), which may contribute to additional variability.

Finally, during the northern winter solstice ($L_{\mathrm{S}} \sim 270^{ \circ}$), both the Mars PCM and the NGIMS data reveal a decrease of the homopause altitude from the southern to northern hemisphere (Fig. 11D). However, while the PCM suggests a rather continuous decrease of the homopause altitude with latitude, the NGIMS data suggest a milder decrease from 60^∘^S to 60^∘^N and a sharp decrease at latitudes >60^∘^N. Nevertheless, based on the synthetic tests explained in the previous paragraphs, we must take with caution such a sharp decrease of the homopause altitude in the polar region.

**Fig. 13:**
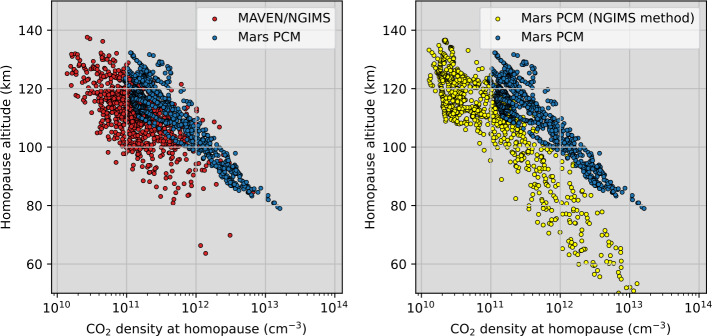
Relation of the homopause altitude and CO_2_ density from MAVEN/NGIMS and the Mars PCM. The left panel shows the comparison of the correlation between the homopause altitude and CO_2_ density from both datasets. For comparison, the right panel shows that correlation but when using the NGIMS retrieval methodology on the Mars PCM data

We may compare as well the CO_2_ densities at the homopause retrieved from the NGIMS data against those predicted by the Mars PCM. The analysis of the NGIMS data, displayed in Fig. 13, shows that, consistent with previous findings, the homopause altitude is not situated at a constant CO_2_ density level, and therefore does not only follow the expansion and contraction of the atmosphere (Slipski et al. 2018). As already highlighted in Sect. 2.3, this is also captured in the Mars PCM simulations (see Figs. 8 and 13). The comparison of the homopause CO_2_ densities reveals that there is an offset between those predicted by the Mars PCM and those from the NGIMS analysis. However, when applying the same NGIMS retrieval methodology to data from the Mars PCM, this bias also appears, suggesting the difference comes from an intrinsic bias in the extrapolation of the CO_2_ density to the homopause in the NGIMS data. Therefore, we conclude that the homopause CO_2_ density from the Mars PCM is generally consistent with the NGIMS dataset.

### Comparison Against TGO/ACS

In this sub-section, we compare the predictions of the homopause characteristics from the Mars PCM against estimates using pressure, temperature and CO_2_ density profiles measured with the ACS instrument aboard ESA’s ExoMars TGO. Belyaev et al. (2022) estimated the turbopause altitude in the martian atmosphere by finding the level at which the eddy and molecular diffusion coefficients are equal. Although related, this definition differs slightly from that of the homopause, which refers to the level where changes in mixing regimes (turbulent eddy mixing vs molecular diffusion) lead to a noticeable variation in atmospheric composition.

Belyaev et al. (2022) calculated the molecular diffusion coefficient $D_{\mathrm{CO}_{2}}$ using the expression given by

1 $$ l_{\mathrm{CO}_{2}}(z) = \left ( Q_{\mathrm{CO}_{2}} \cdot n_{\mathrm{CO}_{2}}(z) \right )^{-1}, $$

2 $$ v_{\mathrm{CO}_{2}}^{th}(z) = \sqrt{ \dfrac{3 \cdot \ k_{B} \cdot T(z) \cdot N_{A}}{M_{\mathrm{CO}_{2}}} }, $$

3 $$ D_{\mathrm{CO}_{2}}(z) = \dfrac{300 \pi \sqrt{2}}{16} \cdot l_{\mathrm{CO}_{2}}(z) \cdot v_{\mathrm{CO}_{2}}^{th}(z), $$

where $l_{\mathrm{CO}_{2}}$ is the free molecular path of CO_2_, $Q_{\mathrm{CO}_{2}}$ is the effective cross section ($Q_{\mathrm{CO}_{2}} = 0.52$ nm^2^), $n_{\mathrm{CO}_{2}}$ is the number density of CO_2_, $v_{\mathrm{CO}_{2}}^{th}$ is the mean molecular thermal velocity, $k_{B}$ is the Boltzmann constant ($k_{B} = 1.38065 \times 10^{-23}$ J K^−1^), $N_{A}$ is Avogadro’s number ($N_{A} = 6.022 \times 10^{23} $ mol^−1^), T is the temperature and $M_{\mathrm{CO}_{2}}$ is the molecular weight of CO_2_ ($M_{\mathrm{CO}_{2}} = 44.01 \mathrm{g}$ mol^−1^) (Jacobson 2005; Mahieux et al. 2012).

In the case of the eddy diffusion coefficient, Belyaev et al. (2022) followed a parameterisation used in some previous studies (Krasnopolsky 2019; Rosenqvist and Chassefière 1995) and given by

4 $$ K(z) = K_{0} \cdot \left ( \dfrac{n(\mathrm{z=35km})}{n(z)} \right )^{-1/2}, $$

where K(z) is the altitude-dependent eddy diffusion coefficient, K_0_ is a constant ($K_{0} = 10^{7}$ cm^2^ s^−1^) and n(z) is the atmospheric number density. This parameterisation of the eddy diffusion coefficient therefore assumes that the $K(\mathrm{z=35km})=K_{0}$ is constant throughout all latitudes and seasons, but not necessarily at higher altitudes, where it will vary following the expansion and contraction of the atmosphere.

Since TGO/ACS simultaneously measures the vertical profiles of p(z), T(z) and $n_{\mathrm{CO}_{2}}(z)$, one can estimate the turbopause altitude by identifying the level at which D=K in each solar occultation observation. However, as with the homopause altitude estimates with MAVEN/NGIMS, this approach relies on assumptions whose uncertainties are difficult to quantify. We therefore use the simulations from the Mars PCM to assess the reliability of this methodology in predicting the altitude of the homopause from other atmospheric parameters.

**Fig. 14:**
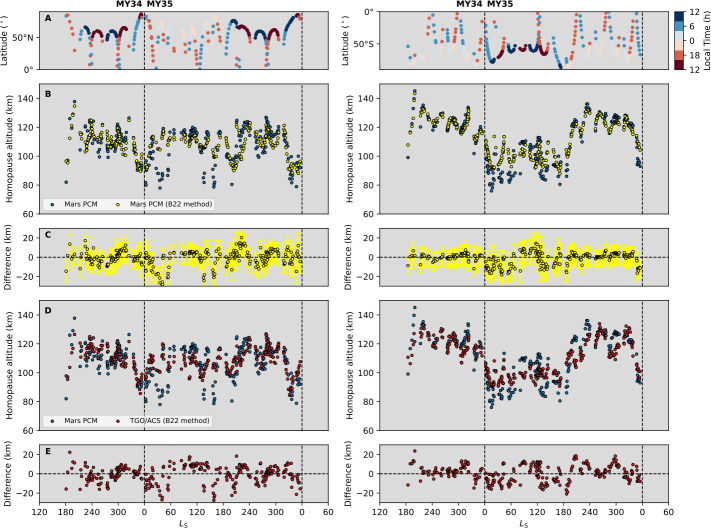
Comparison of the TGO/ACS estimates of the homopause altitude with respect to those from the Mars PCM. The different panels show, for the northern (left column) and southern (right column) hemispheres, the following quantities: A) Latitude and solar longitude of the tangent points of the TGO/ACS occultations, coloured by the local time. B) Homopause altitudes from the Mars PCM (blue points) and the estimated values using the method of Belyaev et al. (2022) (B22 method) using profiles from the same model (yellow points). C) Difference between the two quantities shown in panel B. The error bars indicate the sensitivity of the estimated homopause altitude when multiplying and dividing the value of $K_{0}$ by a factor of two. D) Homopause altitudes from the Mars PCM (blue points) and the estimated values using the B22 method using profiles from TGO/ACS (red points). E) Difference between the two quantities shown in panel D

Figure 14 compares the turbopause altitudes estimated using this approach, applied to both TGO/ACS and Mars PCM profiles, with the nominal PCM homopause altitudes derived from the Ar/N_2_ ratio (see Sect. 2.1). The internal PCM comparison (Fig. 14B,C) allows us to evaluate the accuracy of the methodology used by Belyaev et al. (2022). The results indicate that this approach provides a satisfactory first-order estimate of the homopause altitude, reproducing both the absolute magnitude and the seasonal–latitudinal variability (mean difference 0.2 ± 6.1 km). Nevertheless, local deviations exceeding 20 km occur, particularly outside the southern hemisphere during the second half of the Martian year, where agreement is otherwise satisfactory (<5 km). This is possibly because of the choice of parameterisation for the Eddy diffusion coefficient: the value of $K_{0}= 10^{7}$ cm^2^ s^−1^ is representative of this season and location, but the assumption that it is constant throughout the year introduces some biases when predicting the value of the homopause altitude at other times and locations. The error bars in Fig. 14C represent the change in estimated homopause altitude when assuming values of $K_{0}= 5 \times 10^{6}$ and $2 \times 10^{7}$ cm^2^ s^−1^ instead (i.e., factor of 2). We observe that a factor of 2 in the assumed eddy diffusion coefficient produces a difference in the estimated homopause altitude of approximately 10 km.

A similar pattern is observed when comparing homopause estimates derived from measured TGO/ACS profiles to those predicted by the Mars PCM (Fig. 14D,E). The differences across all points average 1.2 ± 7.9 km, with some larger departures (>20 km) primarily in the first half of the year. These differences are similar to the intrinsic uncertainties of the methodology, and we therefore take the Mars PCM homopause trends to be generally consistent with those inferred from the TGO/ACS data.

## Variability of the Eddy Diffusion Coefficient

The previous two sections examined the variability of the homopause altitude simulated by the Mars PCM and compared it with empirical estimates derived from two different Mars orbiters. In this section, we investigate how this variability can be translated into the definition of an eddy diffusion coefficient suitable for use in 1-D models of the Martian atmosphere. Such 1-D models often adopt simplified parameterisations of transport and turbulent mixing to focus on other aspects of atmospheric physics, such as photochemistry, microphysics, or escape processes. Our objective is to define a parameterisation of eddy diffusion on Mars that enables these 1-D models to reproduce the homopause variability predicted by the Mars PCM.

### Estimation of the Eddy Diffusion Profile and Strength

To estimate the strength of the eddy diffusion, we compare Ar/N_2_ profiles from the Mars PCM with respect to those simulated using a 1-D photochemical model (Alday et al. 2023). Specifically, the diffusion model solves the continuity equation given by

5 $$ \dfrac{\partial n_{i}}{\partial t} = P_{i} - L_{i} - \dfrac{\partial \phi _{i}}{\partial z}, $$

where $n_{i}$ is the number density of a given species in a given atmospheric layer, t and z represent the time and altitude, $P_{i}$ and $L_{i}$ represent the production and loss of density due to chemical reactions, and $\phi _{i}$ is the flux exchange between atmospheric layers. In our case, since we are mostly interested in simulating the relative diffusion of Ar and N_2_, both of which are largely chemically inert, we assume that $P_{i}=L_{i}=0$. The flux is estimated considering both molecular and eddy diffusion following

6 $$ \phi _{i} = - (K + D_{i})\cdot \dfrac{\partial n_{i}}{\partial z} - n_{i} \cdot \left [ K \left ( \dfrac{1}{H_{0}} + \dfrac{1}{T} \dfrac{\partial T}{\partial z} \right ) + D_{i} \left ( \dfrac{1}{H_{i}} + \dfrac{(1+\alpha )}{T} \dfrac{\partial T}{\partial z} \right ) \right ] $$

where K and $D_{i}$ are the eddy and molecular diffusion coefficients, $H_{0}$ is the mean scale height, $H_{i}$ is the gas-dependent scale height, α is the thermal diffusion coefficient, and T is the temperature (see Banks and Kockarts 1973 for derivation of flux equation). In our diffusion model, the molecular diffusion coefficient is parameterised using

7 $$ D(z) = \dfrac{A \cdot T(z)^{s}}{n(z)}, $$

where the coefficients A and s are taken as $A=10^{17}$ and s=0.75 for all species but for H and H_2_, which take the values $A_{\mathrm{H}_{2}}= 2.23 \times 10^{17}$, $A_{\mathrm{H}}= 8.4 \times 10^{17}$, $s_{\mathrm{H}_{2}}=0.75$, $s_{\mathrm{H}}=0.597$ (Cangi et al. 2023; Hunten 1973).

In the case of the eddy diffusion coefficient, one can in principle adopt any values and vertical profile. Using vertical profiles of CO/CO_2_ measured by the Nadir and Occultation for MArs Discovery (NOMAD) instrument aboard ExoMars TGO between 70–105 km, Yoshida et al. (2022) showed that an altitude-dependent eddy diffusion coefficient better reproduced the observed profiles than a vertically uniform one. Specifically, they used a parameterisation of the type $K(z)= \kappa \cdot n(z)^{-1/2}$, which follows the approximation of internal wave activity and conservation of energy density (Lindzen 1971).

In this study, we perform a similar exercise as Yoshida et al. (2022) to define the vertical profile of the eddy diffusion coefficient. We consider three parameterisations for the eddy diffusion profile:

1. The eddy diffusion profile is considered constant with altitude:
  8 $$ K(z) = K_{0} $$
2. The eddy diffusion profile varies as a function of the atmospheric number density following:
  9 $$ K(z)= \kappa \cdot n(z)^{-1/2} $$
3. The eddy diffusion profile is a mixture of the two previous cases, following the derivation in Appendix B:
  10 $$ K(z) = \textstyle\begin{cases} \kappa \cdot n(z)^{-1/2}, & \text{if } n(z) \geq \dfrac{1.3 \times 10^{40}}{\kappa ^{2}}, \\ \dfrac{\kappa ^{2}}{\sqrt{1.3 \times 10^{40}}}, & \text{if } n(z) < \dfrac{1.3 \times 10^{40}}{\kappa ^{2}} \end{cases} $$

In all these parameterisations, the densities are defined in cm^−3^ and the diffusion coefficients in cm^2^ s^−1^.

**Fig. 15:**
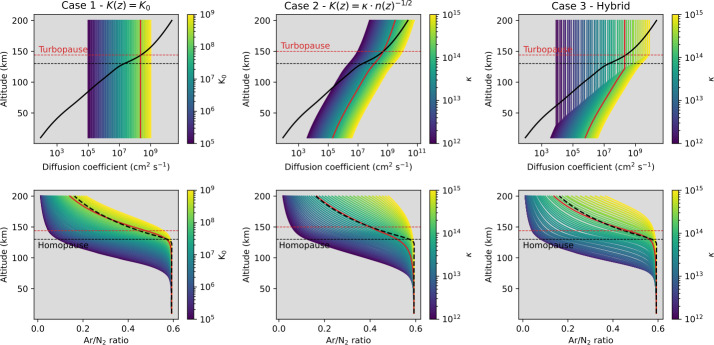
Summary of the methodology used to estimate the eddy diffusion coefficient. Each column represents the method used to derive the strength of the coefficient using three different parameterisations for its vertical structure, as described in the text. Top panels: The black line represents the molecular diffusion profile calculated using the temperature and density profiles from the Mars PCM. The coloured lines represent the eddy diffusion profiles tested in each case, while the red line highlights the profile that best reproduces the Ar/N_2_ profile from the Mars PCM. Bottom panels: The black dashed line represents the Ar/N_2_ profile from the Mars PCM. The coloured lines represent the derived Ar/N_2_ profiles from the 1-D model when using different values of the eddy diffusion profiles, and the red line shows the case that best matches the profile from the Mars PCM. The red and dashed lines represent the derived values of the turbopause and homopause altitudes, respectively

Figure 15 shows an example of the methodology used to define the values of the $K_{0}$ and κ constants for a given profile from the Mars PCM. For all three parameterisations of the eddy diffusion profile, we vary the values of $K_{0}$ and κ and run the 1-D diffusion model until the Ar/N_2_ profile converges to a steady-state solution. Then, we find the profile of the Ar/N_2_ ratio that best matches the one from the Mars PCM based on a minimisation of the root-mean-square error. Finally, we identify the corresponding value of the $K_{0}$ and κ.

When comparing the best-fitting cases for each eddy diffusion profile parameterisation, we can assess how closely they reproduce the PCM results. In contrast to the findings of Yoshida et al. (2022), we find that a constant eddy diffusion profile provides a better match to the Ar/N_2_ profile from the Mars PCM than one following $K(z)= \kappa \cdot n(z)^{-1/2}$. Specifically, the increase of the eddy diffusion profile as $n(z)^{-1/2}$ produces an excessively smooth decrease of the Ar/N_2_ profile with altitude compared to the PCM, which shows instead a sharper change of this ratio at the homopause. This behaviour is best captured by a constant eddy diffusion profile.

It should be noted that, since we are analysing the Ar/N_2_ profiles, our sensitivity to the eddy diffusion parameterisation is limited to the vicinity of the homopause, where this ratio changes owing to the relative strengths of eddy and molecular diffusion. Consequently, we are unable to constrain the eddy diffusion profile at other altitudes. In contrast, the CO/CO_2_ profiles used by Yoshida et al. (2022) constrain the eddy diffusion coefficient at lower altitudes, where the Ar/N_2_ ratio provides little information because the atmosphere is well mixed. To capture both regimes, we therefore derived a third parameterisation of the eddy diffusion profile that is consistent with the calculations of Yoshida et al. (2022) while also providing a good fit to the Ar/N_2_ profile from the Mars PCM (see Fig. 15).

### Variability of Eddy Diffusion on Mars’ Atmosphere

**Fig. 16:**
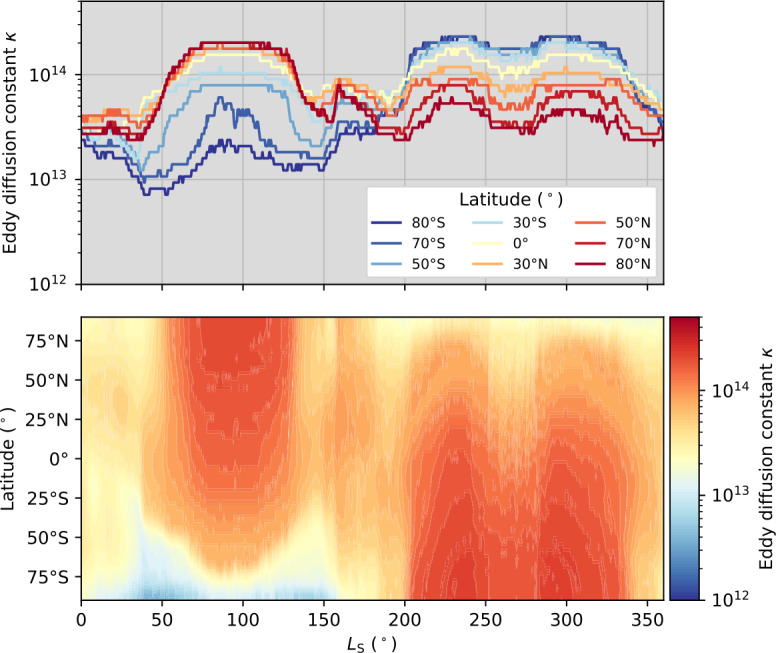
Seasonal and latitudinal variability of the eddy diffusion constant κ. The top panel shows the evolution of this constant at distinct latitudes coloured following the legend. The bottom panel shows similar information, but now shown as a contour plot, where the colour represents the value of the eddy diffusion constant κ

**Fig. 17:**
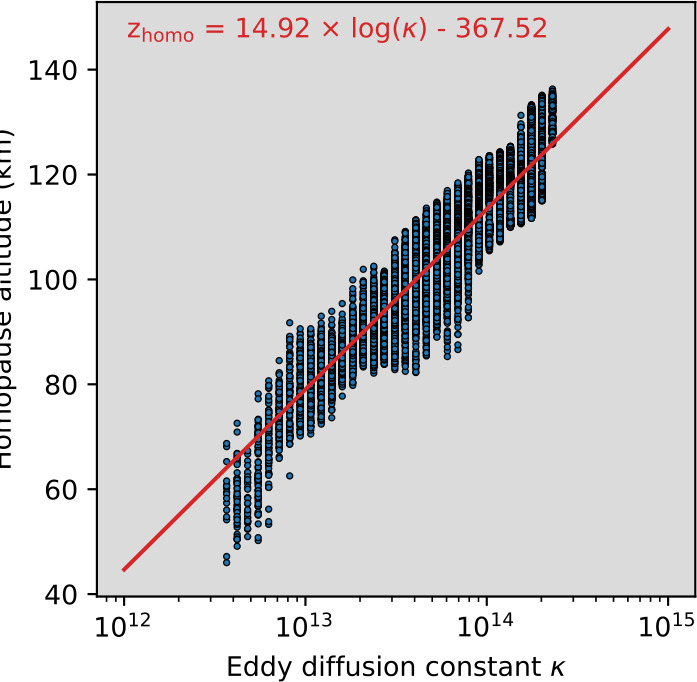
Relationship between the eddy diffusion coefficient κ and the homopause altitude derived from the Mars PCM. The red line indicates the best-fitting linear regression

**Fig. 18:**
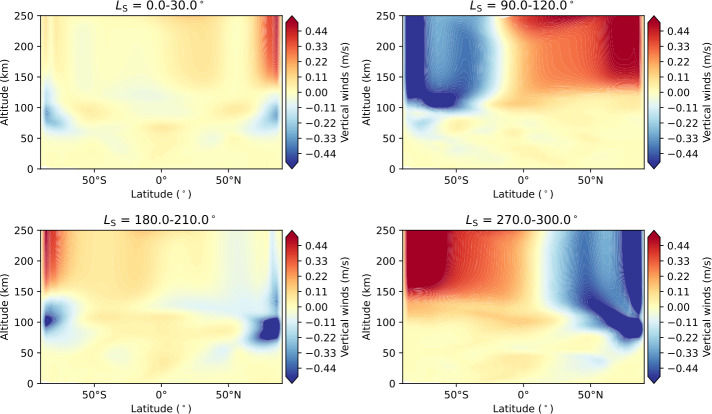
Vertical winds from the Mars PCM averaged across longitude and across the $L_{\mathrm{S}}$ ranges indicated at the top of each panel. Positive values in the colourbar (red) indicate upward winds, while negative values (blue) indicate downward winds

The methodology explained in Sect. 4.1 is applied to the diurnally averaged profiles from the Mars PCM, so that we can investigate the variability of the eddy diffusion coefficient across different latitudes and seasons. Figure 16 summarises the results, revealing spatiotemporal variations of the eddy diffusion coefficient spanning about two orders of magnitude (κ ranging from $4 \times 10^{12}$ to $2 \times 10^{14}$). As expected, these variations are closely correlated with the changes in homopause altitude shown in Fig. 3. The relationship between these two quantities can be approximated through a linear function of the type $z_{\mathrm{homo}} = 14.92 \times \log (\kappa )-367.52$ km (see Fig. 17). The eddy diffusion coefficient peaks globally in the summer hemispheres with values of around $\kappa \sim 2 \times 10^{14}$, while the local maxima during the equinox periods peaks at the equatorial region with values around $\kappa \sim 4 \times 10^{13}$ at $L_{\mathrm{S}} = 0^{\circ}$ and $\kappa \sim 8 \times 10^{13}$ at $L_{\mathrm{S}} = 180^{\circ}$. The global minima is found at high southern latitudes during the first half of the year with values around $\kappa \sim 4 \times 10^{12}$ to $\kappa \sim 1 \times 10^{13}$.

The values of the eddy diffusion coefficient in Fig. 16 might also be compared with the estimations of derived by Yoshida et al. (2022) using the CO/CO_2_ profiles measured with TGO/NOMAD during the solar longitude periods $L_{\mathrm{S}} = 90$–$120^{\circ}$ and $L_{\mathrm{S}} = 240$–$270^{ \circ}$. While a one-to-one comparison requires averaging at the specific locations and times of the TGO occultation points, we can make a first-order analysis by comparing the values derived by Yoshida et al. (2022) with the range of values from the Mars PCM at the selected $L_{\mathrm{S}}$ periods and within a latitudinal range between 40–60^∘^ in both hemispheres (see Table 1). Overall, we observe that the values derived in both studies are consistent with each other, including the expected trends regarding the latitudinal/seasonal variability.

**Table 1:** Comparison of the eddy diffusion constant κ in different latitudes and seasons compared to the values derived by Yoshida et al. (2022), whose uncertainties from systematic biases are estimated to be in the 10–20% range

Region	$L_{S}$ (°)	Yoshida et al. (2022) (*κ*)	This study (*κ*)
North	90–120^∘^	1.25 × 10^14^	(1.5–2.3)×10^14^
South	90–120^∘^	4.25 × 10^13^	(3–9)×10^13^
North	240–270^∘^	7 × 10^13^	(3–9)×10^13^
South	240–270^∘^	1.5 × 10^14^	(1.7–2)×10^14^

Previous studies have used 1-D photochemical models to estimate the seasonal escape of water on Mars, but have often neglected spatiotemporal variations of the eddy diffusion coefficient (Kleinböhl et al. 2024; Stone et al. 2020). Such variability can influence the distribution of atmospheric species at different times and locations, thereby affecting their potential escape to space. Future studies could integrate the eddy diffusion variations derived here to account for the effects of a variable homopause altitude in escape calculations.

## Conclusions

The homopause altitude defines the region of the atmosphere where the effects of turbulence, which tends to keep the atmosphere well-mixed, are overcome by molecular diffusion, which separates chemical species according to their molecular weight. The diffusive separation of chemical species above the homopause has important implications on the distribution of species in the upper atmosphere. Therefore, constraining the variability of this region in the Martian atmosphere is crucial for improving our understanding of the processes that drive atmospheric escape.

The photochemistry and escape of the atmosphere is often studied using models of a single atmospheric column, which do not resolve the full three-dimensional nature of the atmospheric dynamics, in favour of resolving with a greater accuracy these physical mechanisms. In these models, the strength of turbulent mixing, which defines the altitude and density of the homopause region, is typically parameterised using the concept of eddy diffusion.

In this work, we use simulations from the Mars Planetary Climate Model (Mars PCM) to explore the variability of the Martian homopause and the intensity of the eddy diffusion coefficient. The Mars PCM provides a unique framework for this analysis, as it simulates the global atmospheric circulation from the surface to the exosphere and incorporates a wide range of physical and chemical processes, including, crucially, the effects of molecular diffusion in the upper atmosphere. We use the Ar/N_2_ ratio, two non-condensible species that are well mixed in the lower atmosphere, to trace the homopause region, above which the ratio decreases owing to their different molecular weights.

The simulations from the Mars PCM reveal significant variability of the homopause on different timescales. Seasonally, we find the highest homopause altitudes in the summer polar regions (latitudes >60^∘^), with values of around 120–130 km, and minimum values in the southern polar region during winter, with values as low as 60–90 km. The higher homopause altitudes during the solstice periods with respect to equinox reflect the stronger atmospheric circulation during these times. In addition, we find that these variations in altitude do not track a constant pressure level, but instead they span a large range ($10^{-4}$–$10^{-1}$ Pa), consistent with previous findings using data from MAVEN/NGIMS (Slipski et al. 2018).

Regarding the diurnal variability of the homopause, the simulations from the PCM typically reveal variations between 5–15 km, peaking in the afternoon 12–18 h and finding minima during the night. However, on specific latitudinal bands and seasons, the strength of the diurnal cycle can be stronger ($L_{ \mathrm{s}} \sim $ 50, 150, 210, 330^∘^ in the 40–70^∘^ latitude range), or mostly inexistent (polar day or night during solstice periods). These diurnal trends are consistent with previous findings (Slipski et al. 2018; Rao et al. 2025), who found the highest homopause altitudes during noon and the lowest near 5 h.

Finally, the simulations from the Mars PCM reveal that dust events can trigger sudden rises of the homopause altitude of around 10–20 km, depending on the intensity of the event. This is consistent with the findings of Rao et al. (2025) using NGIMS data, which reported homopause altitudes to rise during the growth phase of the 2018 GDS and decrease in the declining phase of the GDS. Apart from the interannual variability induced by these storms, the simulations reveal variability of around 5–10 km around the equinox periods, but little variability in the summer hemispheres near the solstices.

To assess the validity and accuracy of the PCM simulations, we compare the modelled homopause altitudes with estimates derived from Ar and N_2_ density profiles measured by the NGIMS instrument aboard the MAVEN mission (Jakosky et al. 2017; Slipski et al. 2018; Rao et al. 2025). Overall, the Mars PCM reproduces the magnitude and seasonal-latitudinal trends of the homopause altitude, although it appears to underestimate the strength of the diurnal cycle. In addition, using the model to investigate potential uncertainties and biases in the NGIMS measurements, we find that the homopause CO_2_ densities reported by the instrument are likely underestimated.

We also compare the Mars PCM homopause altitudes with estimates derived from pressure, temperature, and CO_2_ density profiles from the ACS instrument aboard the ExoMars TGO (Belyaev et al. 2022), together with a parameterisation of the eddy diffusion and molecular diffusion coefficients. As a first test, we apply this methodology to synthetic PCM data and find that, while it provides satisfactory first-order predictions of the homopause altitude, the assumption of a constant eddy diffusion coefficient at 35 km introduces biases in predicting seasonal and latitudinal variability. Nevertheless, the comparison with TGO/ACS data indicates that the Mars PCM captures the overall seasonal trends of the homopause altitude.

Finally, we use a 1-D diffusion model to constrain the magnitude and variability of the eddy diffusion coefficient, in a manner consistent with simulations from the Mars PCM. Our calculations indicate that the PCM profiles are best reproduced using a constant eddy diffusion coefficient in the vicinity of the homopause. Building on this, we propose a parameterisation that incorporates the $K(z)= \kappa \cdot n(z)^{-1/2}$ variation of the eddy diffusion coefficient suggested in previous studies, but with a saturation at an altitude below the homopause to better match the PCM profiles. Applying this methodology across an entire Martian year, we find that the eddy diffusion coefficient can vary by roughly one to two orders of magnitude (κ ranging from $4 \times 10^{12}$ to $2 \times 10^{14}$). This parameterisation provides a practical tool for future 1-D atmospheric photochemistry and escape models, allowing them to account for the effects of a variable homopause altitude.

## Data Availability

The MAVEN data used to derive the homopause altitude and CO_2_ density at the homopause are publicly available in https://pds-atmospheres.nmsu.edu/data_and_services/atmospheres_data/MAVEN/maven_main.html. The pressure, temperature and CO_2_ density vertical profiles used to estimate the homopause altitudes from the TGO/ACS dataset are available in Belyaev (2022). The Mars PCM, used to estimate the homopause characteristics from simulations of the Ar/N_2_ ratio, is available in https://svn.lmd.jussieu.fr/Planeto/trunk/LMDZ.MARS/. The climatology of the homopause characteristics and eddy diffusion coefficient derived in this study are available in Alday and González-Galindo (2025).

## Appendix A: Vertical Winds from the Mars PCM

The vertical winds in the atmosphere can have an impact in the relative abundances of the Ar/N_2_ ratios in the atmosphere, which in turn impact our derivation of the homopause altitudes (see Sect. 2.1). To provide a more detailed view of the strength of advection in the atmosphere of Mars, Fig. 18 provides a view at the strength of vertical winds around the equinoxes and solstices.

## Appendix B: Derivation of the Eddy Diffusion Coefficient Profile

The comparisons of the simulated Ar/N_2_ from the 1-D model and those from the Mars PCM reveal that near the homopause, the decrease of this ratio is best reproduced when using a constant eddy diffusion profile. On the other hand, Yoshida et al. (2022) revealed that an altitude-varying eddy diffusion profile best explained measurements of the CO/CO_2_ ratio measured from TGO/NOMAD at lower altitudes, between 70–105 km. In this appendix, we aim to derive an eddy diffusion profile that, while following the altitude dependence estimated by Yoshida et al. (2022), provides a good fit of the Ar/N_2_ profile from the Mars PCM.

The eddy diffusion profile from Yoshida et al. (2022) is parameterised as

B.1 $$ K(z)=\kappa \cdot n(z)^{-1/2}, $$

where κ is a constant value. On the other hand, the molecular diffusion coefficient in our 1-D model is parameterised as

B.2 $$ D(z) = \dfrac{A \cdot T(z)^{s}}{n(z)}, $$

where the coefficients A and s are taken as $A=10^{17}$ and s=0.75 for Ar and N_2_. At the turbopause, where both coefficients are equal, we therefore have

B.3 $$ n_{\mathrm{turbo}} = \dfrac{(A \cdot T_{\mathrm{turbo}}^{s})^{2}}{\kappa ^{2}}. $$

Following the estimated characteristics of the homopause from the Mars PCM shown in Fig. 8, while the homopause density spans several orders of magnitude, the temperature variations are much milder. In particular, if we assume $T_{\mathrm{turbo}}=120~\mathrm{K}$, then we get

B.4 $$ n_{\mathrm{turbo}} = \dfrac{1.3 \times 10^{37}}{\kappa ^{2}}. $$

From the comparisons of the 1-D diffusion model simulations and the profiles from the Mars PCM, we typically find the turbopause altitude at 10–20 km above the homopause altitude. Therefore, in order to ensure a constant eddy diffusion profile in the vicinity of the homopause altitude, we fix a uniform value of K for $n(z) \leq 10^{3}\cdot n_{\mathrm{turbo}}$, with a value of

B.5 $$ K = \dfrac{\kappa ^{2}}{\sqrt{1.3 \times 10^{40}}}. $$

Combining both regimes of the eddy diffusion coefficient variation with altitude, we end up with

B.6 $$ K(z) = \textstyle\begin{cases} \kappa \cdot n(z)^{-1/2}, & \text{if } n(z) \geq \dfrac{1.3 \times 10^{40}}{\kappa ^{2}}, \\ \dfrac{\kappa ^{2}}{\sqrt{1.3 \times 10^{40}}}, & \text{if } n(z) < \dfrac{1.3 \times 10^{40}}{\kappa ^{2}} \end{cases} $$
